# The Pattern and Distribution of Induced Mutations in *J. curcas* Using Reduced Representation Sequencing

**DOI:** 10.3389/fpls.2018.00524

**Published:** 2018-04-23

**Authors:** Fatemeh Maghuly, Stephan Pabinger, Julie Krainer, Margit Laimer

**Affiliations:** ^1^Plant Biotechnology Unit, Department of Biotechnology, Vienna Institute of BioTechnology, University of Natural Resources and Life Sciences, Vienna, Austria; ^2^Molecular Diagnostics, Center for Health & Bioresources, Austrian Institute of Technology, Vienna, Austria

**Keywords:** chemical mutagenesis, SNP calling, crop improvement, biofuel, reverse genetic

## Abstract

Mutagenesis in combination with Genotyping by Sequencing (GBS) is a powerful tool for introducing variation, studying gene function and identifying causal mutations underlying phenotypes of interest in crop plant genomes. About 400 million paired-end reads were obtained from 82 ethylmethane sulfonate (EMS) induced mutants and 14 wild-type accessions of *Jatropha curcas* for the detection of Single Nucleotide Polymorphisms (SNPs) and Insertion/Deletions (InDels) by two different approaches (nGBS and ddGBS) on an Illumina HiSeq 2000 sequencer. Using bioinformatics analyses, 1,452 induced SNPs and InDels were identified in coding regions, which were distributed across 995 genes. The predominantly observed mutations were G/C to A/T transitions (64%), while transversions were observed at a lower frequency (36%). Regarding the effect of mutations on gene function, 18% of the mutations were located in intergenic regions. In fact, mutants with the highest number of heterozygous SNPs were found in samples treated with 0.8% EMS for 3 h. Reconstruction of the metabolic pathways showed that in total 16 SNPs were located in six KEGG pathways by nGBS and two pathways by ddGBS. The most highly represented pathways were ether-lipid metabolism and glycerophospholipid metabolism, followed by starch and sucrose metabolism by nGBS and triterpenoid biosynthesis as well as steroid biosynthesis by ddGBS. Furthermore, high genome methylation was observed in *J. curcas*, which might help to understand the plasticity of the *Jatropha* genome in response to environmental factors. At last, the results showed that continuously vegetatively propagated tissue is a fast, efficient and accurate method to dissolve chimeras, especially for long-lived plants like *J. curcas*. Obtained data showed that allelic variations and *in silico* analyses of gene functions (gene function prediction), which control important traits, could be identified in mutant populations using nGBS and ddGBS. However, the handling of GBS data is more difficult and more challenging than the traditional TILLING strategy in mutated plants, since the *Jatropha* genome sequence is incomplete, which makes alignment and variant analysis of target sequence reads challenging to perform and interpret. Therefore, providing a complete *Jatropha* reference genome sequence with high quality should be a priority for any breeding program.

## Introduction

*Jatropha curcas* (family *Euphorbiaceae*) originates from Central America and currently is distributed throughout all tropical and subtropical regions. It could be one of the most valuable crops, not only as a biofuel plant but also due to the high protein amount in its seeds, which make it attractive as animal feed and organic manure. Beside its variable toxin content, consisting mainly of diterpenes (phorbol esters) and ribosome-inactivating proteins (curcin) (Maghuly et al., 2013, 2016), the plant raises interest for its ability to produce pharmaceutically important compounds. However, the genome of *J. curcas* is highly homogenous (*C* = 0.416 × 109 bp, 2n = 22), and its genetic diversity is low (Carvalho et al., 2008; Maghuly et al., 2015; Maghuly and Laimer, 2017). Compared to other oilseed crops, research and development of *J. curcas* is still at a very early stage (King et al., 2013), however, the release of the whole genome sequence of *J. curcas*^1^ opened a new era in genomic research (Sato et al., 2011; Zhang et al., 2014; Wu et al., 2015). Nevertheless, data from genome projects alone will not automatically offer solutions for increasing productivity, decreasing toxin amounts and detailed understanding of pathogen resistance. Still, detailed information of the genetic landscape and gene function is a premise to (a) uncover why some accessions of *J. curcas* produce toxins and others not, (b) why – with their narrow genetic variation – *J. curcas* can grow in such diverse environmental conditions, (c) know, how biochemistry contributes to increases in oil yield and quality, (d) understand the processes of growth and development.

Induced mutations are useful to study gene functions in populations with a uniform genetic background, like *J. curcas*, as their genetic variation is typically dissolved (Henry et al., 2014). Further, chemical and physical mutagenesis has a long history for plant breeding to identify and select desirable traits (e.g., oil quality and low toxicity) for crop improvement, and is shown to be an effective method to induced mutagenesis in a wide range of plants (Bado et al., 2015). These methods provide necessary population resources, structural variation, and the identification of gene functions by gene knockouts, disruptions, and missense. Physical mutagenesis could produce huge DNA alterations such as large deletions, from 1 to 230 kbp, with frame-shifts or total gene loss, inversions, translocations and small intragenic mutations, while chemical mutagenesis mainly causes point mutations (base substitutions), small insertions and deletions (Mba et al., 2010). Compared to various type of chemical mutagenesis, ethyl methanesulfonate (EMS), an alkylating agent, offers an efficient and potent approach, creating single base pair changes or SNPs by acting primarily on guanine residues. These SNPs may either occur in coding regions, thus changing the encoded amino acids (non-synonymous), be silent (synonymous) or occur in non-coding regions, where they may induce changes in the promotor sequences or regulatory regions as well as intergenic regions without representing any measurable phenotype (Sikora et al., 2011). Non-synonymous SNPs can be used as markers for association studies to detect genetic variations related to phenotypic traits. They may also influence gene expression (up- or down regulation of gene transcription), and therefore represent a valuable tool for plant improvement (Ng and Henikoff, 2001).

Significant efforts toward large-scale characterization, screening, identification and discovery of SNP-mutants, e.g., DNA chips and microarrays and Targeting Induced Local Lesions In Genomes (TILLING) in combination with Next Generation Sequencing (NGS) have been used with success in crop plants (Till et al., 2007; Tsai et al., 2011; Maghuly et al., 2015, 2016, 2017). However, techniques that use quantitative polymerase chain reaction (PCR) only work for small fragments of DNA, array platforms are expensive and not flexible, and although sequencing candidate genes is the most accurate and straightforward alternative, it is relatively expensive (Sidhu et al., 2015). On the other hand, several genome-wide genotyping technologies have been developed and commercialized for SNP allele calling, e.g., Whole Genome Sequencing (WGS), RNA Sequencing (RNA Seq), exome capture, Restriction-site Associated DNA Sequencing (RADSeq), and Genotyping By Sequencing (GBS). RADSeq and GBS depend on Restriction Enzyme (RE) digestion. In contrast to WGS, they represent efficient and cost-effective methods for a large number of individuals to produce genetic information by applying efficient pooling strategies and reducing the representation of the genome (Beissinger et al., 2013). Further, RADSeq focusses on high target coverage of DNA fragment sequencing, while GBS involves sequencing DNA fragments at low target coverage and minimizing reads in repetitive sequences (Beissinger et al., 2013). The fact that GBS avoids random shearing and does not need size selection, nor specific library preparation, is completed in only two steps on plates followed by PCR amplification of the pooled library and is very simple compared to RADSeq, makes it a cost-effective alternative (Chen et al., 2013). In fact, low coverage genotyping methods produce large proportion of missing data (Wickland et al., 2017). The nGBS approach represents the genomic sequence between restriction sites by a single RE (Elshire et al., 2011), while double digested GBS (ddGBS), an alternative method to GBS, uses two enzymes, which can generate a suitable and uniform complexity reduction (Poland et al., 2012). Since the selection of optimal REs is critical for this method, some previous reports applied whole genome sequence data for *in silico* prediction of ddGBS to identify the size, number and genome position of digested fragments (Shirasawa et al., 2016).

However, besides different methods for identifying the effective mutation, it is of utmost necessity to use screening techniques for dissociation of chimeras from stable mutants. For somatic mutations, few methods exist for isolation of induced mutation or to produce plants without chimeric structure (Forster et al., 2012). Among them, *in vitro* culture techniques like protoplast, cell-, tissue- and organ- culture appear to be a fast, direct and robust method for isolating mutations (Suprasanna and Nakagawa, 2012). However, in vegetatively propagated plants, several cycles of regeneration are required to reduce the chimeras. If the optimal number of *in vitro* sub-culture was not achieved, the mutant would be chimeric, and the mutation of interest could be lost through subsequently multiplications (Shu et al., 2012).

In the current study, the development of a population of chemically induced mutants of *J. curcas* is described, which was screened for SNPs and InDels and subjected to functional analysis studies. To screen for rare mutations in genes of interest and to understand *in silico* gene function prediction on a whole-genome scale, two GBS based methods, namely normalized (n)GBS and double digest (dd)GBS were compared on 82 mutants and 14 wild-type accessions of *J. curcas.* Results show that nGBS-analyses of *J. curcas* mutants could be used as a reverse genetic method for genome-wide discovery of SNP loci even in crops with low genetic diversity.

## Materials and Methods

### Mutant Population

A mutant population of 1,000 seeds from a selected *J. curcas* tree originated from Ethiopia was generated using three concentrations (0.4, 0.8, and 1.6%) of the chemical mutagen ethyl methanesulphonate (EMS) for 3 different durations (0.5, 1.5, and 3 h) (Maghuly et al., 2017). The mutated population (M_1_V_1_) was transferred to appropriate tissue culture media and kept in a growth chamber with 12 h light at 28°C + - 2°C (da Câmara Machado et al., 1997; Maghuly et al., 2017), at the PBU, BOKU University, Vienna, Austria. Control samples, not exposed to EMS treatment were grown in the same conditions. To reduce chimeras, subcultures were repeated every 3 weeks by the division of apical and lateral meristems. Following this process, the mutant lines from M_1_V_6_ were subjected to genotypic analyses. Out of them, 96 samples containing 14 non-mutated wild-type *J. curcas* accessions and 82 mutated accessions were selected for the following studies (**Supplementary Table S1**).

### DNA Extraction

100 mg of *in vitro* fresh young leaf tissue of each M_1_V_6_ plants was used to extract total genomic DNA using DNeasy Plant Mini kit (Qiagen) following the supplier’s instructions. The quality and concentration of DNA of each sample were determined using both gel electrophoresis and spectrophotometry.

### Normalized Genotyping by Sequencing (nGBS) Using *MslI*

Genomic libraries were prepared following the protocol described by Elshire et al. (2011). Two hundred nano gram of each genomic DNA was digested with 1 Unit RE *MslI* (New England Biolabs, NEB) and 1 × NEB buffer 4 for 1 h at 37°C in 30 μl volumes. Incubation at 80°C for 20 min inactivated the REs.

The Encore Rapid Multiplex System was used to prepare Indexed Illumina libraries in a 96-well PCR plate by mixing 15 μl of each digested DNA with 3 μl of L2 Ligation adaptors, and 12 μl master mix (4.6 μl D1 water, 6 μl L1 Ligation Buffer, 1.5 μl L3 Ligation Enzyme). The reaction of ligation was completed at 25°C for 15 min. Next, 20 μl Final Repair Master Mix was added to each well, and the reaction was incubated at 72°C for 3 min.

Fifty microliter of TE buffer (10 mM Tris/HCl, 50 mM EDTA, pH 8) were added in tubes with reactions, which mixed with 80 μl Agencourt XP beads (Beckman Coulter), incubated for 10 min at RT and placed the tubes on a magnet to collect the beads after 5 min. The beads were washed two times with 200 μl 80% Ethanol, after discarding the supernatant. Beads were air dried for 10 min, and libraries were eluted in 20 μl Tris buffer (5 mM Tris/HCl, pH 9). Ten microliter of each library were amplified in 20 μl PCR reactions using MyTaq (Bioline) and standard Illumina TrueSeq amplification primers. The cycle number was limited to 10 cycles.

Five microliter of all 96 amplified libraries with different adaptors were pooled, and PCR primers, as well as small amplicons, were removed by 0.6 volume of beads using Agencourt AMPure XP PCR purification kit (Beckman Coulter). Further, the PCR enzyme was removed by an additional purification step on MinElute column (Qiagen). The pooled libraries were eluted up to 20 μl by Tris Buffer (5 mM Tris/HCl, pH 9).

One microgram pooled GBS library was normalized using the Trimmer Kit (Evrogen) by mixing 12 μl water and 4 μl of 4 × hybridization buffer. The prepared mix was denatured at 98°C for 3 min and renaturated at 68°C for 5 h; after that 15 μl of 2 × DSN buffer was added and continued with incubation at 68°C for 10 min. The reaction was treated with one Unit of DSN enzyme (1U/μl) to degrade double-stranded DNA and incubated at 68°C for 30 min. The reaction was terminated using 20 μl DSN Stop Solution, purified on a Qiagen MinElute column and eluted in 10 μl Tris Buffer (5 mM Tris/HCl pH 9).

The Emulsion PCR (emPCR) was carried out for library re-amplification using buffers and enzymes from the emPCR Kit (Roche 454). The oil-surfactant mixture for creating the emulsion, columns and buffers for post-emPCR purification were provided by the Micellula DNA Emulsion & Purification Kit (EURx). PCR water phase was produced by 5 μl normalized DNA, 20 μl of 5 × Amp Mix, 40 μl of emPCR Additive, 20 μl of BiostabII PCR Enhancer (Sigma), 4 μl of BSA (10 mg/ml, New England Biolabs, NEB), 4 μl of TruSeq primer (50 pM/μl each), 5 μl of emPCR enzyme Mix and 0.2 μl of Ppiase. An oil surfactant phase was created by 440 μl of Emulsion Component T-1, 40 μl of Emulsion Component T-2 and 120 μl of Emulsion Component T-3. One hundred microliter of PCR water phase and 600 μl of oil surfactant phase were mixed in a 1.5 ml reaction tube and vortexed for 5 min to generate a stable emulsion. Hundred microliter of the mix were used for PCR amplification. PCR-cycling conditions consisted of 93°C for 1 min and continued by 18 cycles of 93°C for 30 s, 60°C for 30 s and 68°C for 2 min. The breaking of the emulsion and subsequent DNA purification was done according to the Micellula DNA Emulsion & Purification Kit manual (EURx). The nGBS library was finally size selected (200–500 bp) on a LMP-Agarose gel.

### Double Digest GBS (ddGBS) Library Construction Using *Pst I* and *Msp I*

The ddGBs was performed as described by Poland et al. (2012). 200 ng of genomic DNA were digested with 10 U HF-*PstI* (High Fidelity) and 8 U *MspI* (New England Biolabs, NEB) in 20 μl reaction volume of 1 × NEB buffer 4 for 2 h at 37°C, followed by inactivation of the enzymes at 65°C for 20 min.

The ligation reaction was carried out using 1 × NEB buffer 4, ATP, respective Adapter as described by Poland et al. (2012), containing a mix of common Y shaped adapter and sample specific barcoded adapter and T4 DNA Ligase. The ligation was carried out at 22°C for 2 h and enzyme was inactivated at 65°C for 20 m.

Reactions were diluted with 50 μl TE buffer (10 mM Tris/HCl, 50 mM EDTA, pH 8.0) and mixed with 80 μl Agencourt AMPure XP beads (Beckman Coulter), incubated for 10 min at RT and placed for 5 min on a magnet to collect the beads. After discarding the supernatant, the beads were washed two times using 200 μl of 80% Ethanol. Beads were air dried for 10 min, and libraries were eluted in 10 μl Tris Buffer (5 mM Tris/HCl pH 9).

Three microliter of each library were separately amplified in 20 μl PCR reactions using Herculase II (Agilent Technologies). Cycle number was limited to 14 Cycles.

All 96 amplified samples were pooled, mixed, and purified using MinElute PCR Purification Kit (Qiagen). Concentration was measured on Qubit, and the quality checked on an Agilent High Sensitivity DNA Chip. The GBS library was finally size selected (200–500 bp) on a LMP-Agarose gel.

### Data Analysis, Read Preprocessing, GBS Alignment and SNP Discovery

The libraries were sequenced using Illumina HiSeq 2000 and paired-end sequence reads from FASTQ files were demultiplexed with Illumina’s CASAVA data analysis software and software developed by LGC Genomics. All the raw sequencing reads were deposited in the Short Read Archive (SRA) database is retrievable under the accession number SRP136238 in the SRA database of NCBI.

Reads were discarded using the following criteria (a) reads containing unspecified nucleotides (Ns), (b) reads with a final length of less than 20 bases and (c) reads where the 5′ end did not match with the RE site. Reads were quality trimmed at 3′ end and reads with a final length less than 64 bases were discarded. For all FASTQ files, FastQC reports were created. BWA version 0.7.5a^2^ was used to map (aln/sampe) trimmed reads against the reference genome of *J. curcas* JAT r4.5.^3^ Variant discovery and genotyping of samples was performed with Freebayes v0.9.9^4^ using specific parameters “–min-base-quality 10 –min-supporting-allele-qsum 10 –read-mismatch-limit 3 -min-coverage 5 –min-alternate-count 4 –report-genotype-likelihood-max –exclude-unobserved-genotypes –genotype-qualities –no-mnps –no-complex –ploidy 2.”

Genotype calls were filtered with the following GBS-specific filter rule set: (a) including only variants for which both alleles have been called, (b) genotypes must have been observed in at least 66% of the samples, (c) minimum total depth across all samples must exceed 20 reads, (d) minimum allele depth across all samples must exceed 10 reads, (e) sample genotype data where total read count was below 8 were removed, (f) heterozygous calls were reverted to homozygote calls when the allele balance was outside 20–80%.

Variants with only heterozygous calls were excluded. Annotation of variant effect on genes and transcripts was performed using SnpEff version 3.2^5^ and the Blast2GO annotation tool (Conesa et al., 2005). The predicted genes and transcripts according to the genome annotation were used to predict downstream functional effects of the SNPs.

### Reference Sequences

Sequence and annotation of *J. curcas* JAT r4.5^3^ were used for alignment, and a manually improved GTF file (derived from the provided GFF file) was used with SnpEff to annotate putative functional effects of the variants.

## Results

To screen for novel mutations in genes of interest and to understand *in silico* gene function prediction on a whole-genome scale, nGBS and ddGBs based on Illumina sequencing (by LGC Genomics) were used on 82 mutants and 14 wild-type accessions of *J. curcas*.

To make GBS libraries of *J. curcas*, containing a small size genome (1C = 416 Mb), and to maximize the desired size distribution (200–500 bp) of restriction fragments for sequencing library, different REs were selected. The GBS libraries were prepared and sequenced from the same set of DNAs using M*sl*I for nGBS and P*st*I/M*sp*I for ddGBS with 10, 6 and 4-base recognition sites, respectively.

### Structure and Functional Annotation of nGBS-Based Variants

**FIGURE 1:**
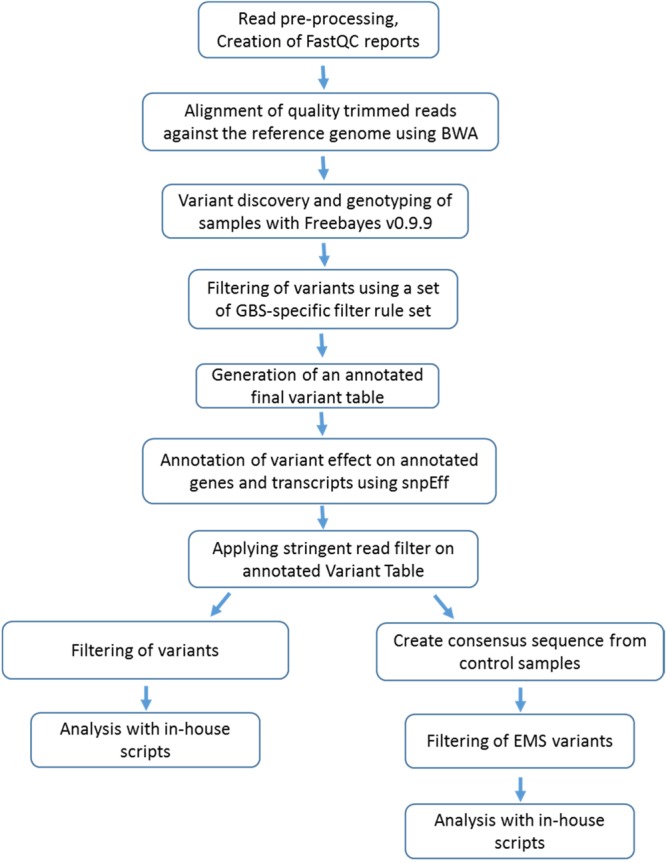
Bioinformatics workflow that was used in this study.

Genomic DNA digested with *MslI* and fragments in the desired size range was recovered in libraries from 96 different individuals tagged with 96 barcodes. Sequencing of 96 samples on Illumina HiSeq 2000 platforms produced 263,340,722 of 100-bp paired-end raw reads. **Figure 1** describes all steps carried out for reading processing. After the low-quality reads were trimmed and filtered, a total of 159,380,823 high-quality reads remained. The average number of reads per individuals was 1,656,035 million, ranging between 49,564 (*Msl*I-D06) and 2,459,394 million reads (*Msl*I-E09). The number of reads among EMS mutant plants ranged from 49,564 (*MsI*I-D06) to 2,459,394 (sample *MsI*I-E09), whereas the number of reads for the wild-type *J. curcas* ranged from 1,195,496 (*MsI*I-H03) to 2,418,812 (*MsI*I-G03) (**Supplementary Table S2**). The provided sequencing reads were mapped to the publicly available reference sequence^3^. Further, additional stringent filters were applied for variant analysis, which removed variations with a total read count less than 20 and a minor allele count less than 10.

Data analyses among 96 *J. curcas* samples identified 79,688 SNPs (62,418 heterozygous), 1,567 InDels and 155,831 missing alleles. The InDels ranged from 2 to 5 bp, with the majority of mononucleotide InDels (**Supplementary Table S3**).

The total number of heterozygous and homozygous SNPs for alternative alleles per variant ranged from 184 (*MsI*I-G11) to 1,059 (*MsI*I-E09) and from 29 (*MsI*I-G11) to 310 (*MsI*I-E09), respectively. Due to the small number of reads, the sample *MSI*I-D06 was removed (**Supplementary Tables S2, S4**).

Further, 1,366 variants were found in coding regions, 421 in introns, 1,541 in intergenic regions, and 1,874/1,723 were located in up- and downstream regions, respectively. The functional effect of SNPs and InDels in genic regions on 945 annotated genes (predicted protein sequences) of the reference genome were classified by the SnpEff program^6^. Among them, 771 were non-synonymous mutations, 507 synonymous, whereas only a small fraction of mutations (18) induced frame-shifts. Of these, we could identify a few causing a loss of start and stop (3 and 7 respectively), and a somewhat higher occurrence in stop gained (60). Further, 8 effects were found in splice site donor and 9 in splice site acceptor (**Table 1**). The details regarding functional annotation of the relevant gene derived non-synonymous SNPs are shown in **Table 1**.

**Table 1:** Genomic features (classification) of SNP and InDel distributions identified by nGBS.

Effect	SNP and InDel occurrences	Number of distinct genes
Synonymous	507	366
Non-synonymous	771	501
Stop gained	60	52
Stop lost	7	7
Start lost	3	2
Frame-shift	18	17
Intron	421	0
Intergenic	1,541	0
Upstream	1,874	1,268
Downstream	1,723	1,143
Splice site acceptor	9	3
Splice site donor	8	5

**FIGURE 2:**
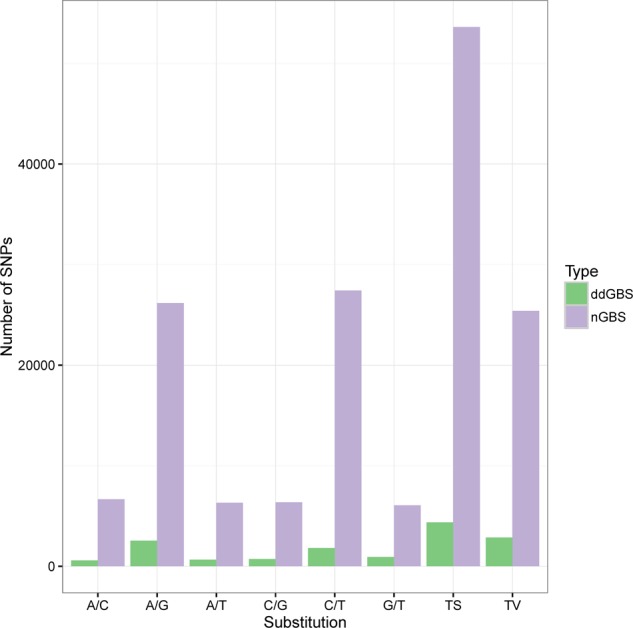
Number of transitions and transversions identified by nGBS and ddGBS.

Depending on the type of nucleotide substitutions, SNPs were classified into transitions (Ts) and transversions (Tv), using VCFtools (**Table 2**). Most of the SNPs were Ts (53,621), with the C/T (51%) and G/A (49%) transitions accounting for 67.8% of the SNPs. The other four SNP types were Tv (25,406), which includes C/G (25%), G/T (24%), C/A (26%) and A/T (25%) transversions accounting for 32.2% of all SNPs. The Ts/Tv ratio in this study was 2.1 (**Figure 2**_nGBS and **Table 2**).

**Table 2:** Number of transitions and transversions identified by nGBS.

Substitution	Number of SNPs
Transitions	53,621
C/T	27,438
A/G	26,183
Transversions	25,406
C/G	6,366
A/T	6,321
A/C	6,660
G/T	6,059
Ts/Tv ratio	2.1

In category Biological Process (BP), functional annotation in genic regions of variable alleles of 235 genes with severe effects were classified in 19 different GO pathways, of which a significant portion was annotated as genes performing DNA integration (335 SNPs and 9 InDels) and protein phosphorylation (13 SNPs). Of the 397 high impact variations, 388 SNPs and 9 InDels were identified, which contain 356 SNPs with non-synonymous effect, 29 stop-gained, 9 frame-shift and 3 stop-lost.

By category, Molecular Function (MF), 41 GO pathways containing 490 SNPs and 13 InDels related to 303 genes were annotated, the highest number of variable alleles belong to the nucleic acid binding and zinc ion binding (386 non-synonymous, 30 stop-gained, 2 stop-lost, and 13 InDels). Out of 503 mutations (SNPs and InDels), 452 were annotated to have a non-synonymous, 34 a stop-gained, 3 a stop-lost and 13 a frame-shift effect. The least GO terms (8) were found under the category Cellular Component (CC) with 10 genes containing 17 SNPs. Out of them, 16 mutation changes showed non-synonymous, and one-stop gained effect (**Supplementary Figure S1** and **Supplementary Table S5**).

To reconstruct the metabolic pathways, the variable alleles in genic regions were mapped to KEGG pathways (Sidhu et al., 2015). In total 16 SNPs (14 with non-synonymous and 2 with stop-gained effects) were located on six KEGG pathways (**Supplementary Table S6**). The most highly represented pathways were ether-lipid metabolism and glycerophospholipid metabolism each with 5 SNPs affecting the same gene (Jcr4S00177.210), followed by starch and sucrose metabolism with 3 SNPs on three different genes. These results showed that identified regions near variable changes could modify the oil and polysaccharide contents, which can be evaluated by various metabolic analyses.

### Detection of EMS-Induced Changes by nGBS

To identify SNPs that were introduced by EMS we compared the non-mutated accessions to the mutated samples. Due to the heterogeneous genetic background of the 14 non-mutated accessions, we implemented a novel approach to build a consensus sequence. The consensus sequence based on 14 wild type *J. curcas* accessions was predicted using the variants that all 14 accessions had in common leading to a reduction of genomic positions to 365. Here 340 consensus sequences are homozygous to the reference and 25 are homozygous to the alternate allele. The final consensus sequence was used for direct comparison of mutant and non-mutant accessions.

Using this consensus sequence, 365 distinct genomic positions could be used for comparison totaling 29,930 positions amongst all 82 mutated samples.

Genotypes varying from the consensus were defined as an EMS-induced mutation. In total 1,014 EMS induced mutations were identified whereas 27,810 positions had the same alleles/SNPs as the consensus sequence. The remaining 1,106 positions were removed because of low read count or missing data (**Supplementary Tables S7, S8**). Overall, SNP changes were distributed in 427 annotated genes. In total, 207 EMS-induced mutations were identified in coding regions and 267 SNPs in intergenic regions followed by 74 in upstream, 29 in downstream and 45 in intron regions.

For individual plants, the number of positions equal to the consensus sequence ranged from 214 in *MSI*I-G11 to 359 in *MSI*I-C09. Of the EMS mutational changes, the smallest number of SNPs was found in sample *MSI*I-B03 with 5 sequence changes, followed by *MSI*I-C08 with 6 changes. The highest number of EMS-induced SNPs was observed in sample *MSI*I-C09 with 38 changes, followed by *MSI*I-C12 with 28 changes (**Supplementary Table S8**). Interestingly, the samples with highest and lowest EMS mutations are related to the treatment group with 0.8% EMS and 1.5 h.

Functional annotation of SNPs in coding regions with high to moderate effects (non-synonymous, stop lost, stop gained, start lost, frame-shift) revealed 40 SNPs in 30 different genes with 38 non-synonymous and 2 stop-gained effects involved in two separate pathways (DNA integration and intracellular protein transport) of BP category (**Supplementary Figure S2** and **Supplementary Table S9**).

In the category MF, 53 SNPs were located in 42 different genes with 2 stop-gain and 40 non-synonymous effects involved in 8 different pathways. The largest proportion of SNPs are effecting the nucleic acid binding and zinc ion binding pathways. Further, the only SNP in CC category effecting the “clathrin adaptor complex; Golgi apparatus” pathway was identified in sample *MSI*I-E01 (**Supplementary Figure S2** and **Supplementary Table S9**).

### Structure and Functional Annotation of ddGBS-Based Variants

Genomic DNA of 96 different individuals was double-digested with REs *Pst*I and *Msp*I and fragments in a size range of 200–500 bp and sequenced on Illumina HiSeq 2000 platform. Sequencing of 96 samples yielded 135,531,226 raw reads. After trimming and filtering the low-quality reads, 118,258,489 high-quality trimmed reads were obtained. The average number of high quality reads per individuals was 1,231,859 reads ranging between 171,348 (*Pst*I-*Msp*I-A04) and 1,798,892 (*Pst*I-*Msp*I-G06) reads. The number of reads among EMS mutant plants ranged from 309,172 (*Pst*I-*Msp*I-A10) to 1,798,892 (*Pst*I-*Msp*I-G06), whereas the number of reads for the non-mutated plants ranged from 171,348 (*Pst*I-*Msp*I-A04) to 1,603,586 (*Pst*I-*Msp*I-G04) (**Supplementary Table S10**).

The produced sequence reads were mapped to reference sequences generated from the publicly available data^7^ (**Supplementary Table S11**). In the 96 *J. curcas* samples, 5,525 heterozygous SNPs and 623 InDels were identified. Also, 3,911 were homozygous to the reference, 1,794 were homozygous to the alternate alleles, and 8,787 were missing. The InDels ranged from 2 to 4 bp, with the majority of mononucleotide InDels. The identified SNPs and InDels in genic regions were distributed across 68 genes (**Table 3**). The total number of heterozygous, homozygous for alternative alleles SNPs and InDels per genomic positions ranged from 15 (*Pst*I-*Msp*I A10) to 101 (*Pst*I-*Msp*I E09) and 4 (*Pst*I-*Msp*I A04) to 26 (*Pst*I-*Msp*I D04, *Pst*I-*Msp*I G07), respectively. These EMS mutated samples originated from different EMS concentrations, i.e., 0.8% 3 h (*Pst*I-*Msp*I A10, *Pst*I-*Msp*I E09, *Pst*I-*Msp*I G07) and 1.6% 3 h (*Pst*I-*Msp*I D04) (**Supplementary Table S12**).

**Table 3:** Genomic features (classification) of SNP and InDel distributions identified by ddGBS.

Effect	SNP and InDel occurrences	Number of distinct genes
Synonymous	30	27
Non-synonymous	54	40
Stop lost	1	1
Codon insertion	1	–
Intron	60	–
Intergenic	58	–
Upstream	101	72
Downstream	104	78
Splice site acceptor	1	1

Further, 60 SNPs and InDels were found in introns, 58 in intergenic and 86 in coding regions (**Table 3**). Of the changes affecting the coding regions, 30 were synonymous and 54 non-synonymous, which were identified in 27 and 40 genes, respectively (**Table 3**). They had very low occurrences in splice site acceptors and stopped lost (a single mutation for each), and higher occurrences in down- and upstream regions (104, 101, respectively).

Most of the identified SNPs were transitions (4,360), with the C/T (42%) and G/A (58%) transitions accounting for 60.4% of the SNPs. On the other hand, 2,859 transversions were identified, which include C/G (25%), G/T (32%), C/A (20%) and A/T (23%) accounting for 39.6% of all SNPs (**Figure 2** _ddGBS and **Table 4**). The Ts/Tv ratio in these samples was 1.5.

**Table 4:** Number of transitions and transversions identified by ddGBS.

Substitution	Number of SNPs
Transitions	4,360
C/T	1,816
A/G	2,544
Transversions	2,859
C/G	715
A/T	651
A/C	574
G/T	919
Ts/Tv ratio	1,5

Functional annotation categorized 22 non-synonymous SNPs located on 16 genes in 6 GO pathways of BP. In the category MF, 23 GO pathways with 42 SNPs on 31 genes were identified, containing 41 non-synonymous and one-stop lost. The lowest number of SNPs (6) were found in category CC, with 4 GO pathways and five genes.

In the category BP, the highest number of SNPs (10) were involved in oxidation-reduction process, followed by 7 SNPs related to protein phosphorylation, while in the MF category 9 SNPs in five genes were also involved in oxidoreductase activity, performing with integration or reduction of molecular oxygen, iron and heme binding, followed by 4 SNPs in 3 genes associated with transferase activity. The type of variation, the location on genes and samples containing are described in the **Supplementary Figure S3** and **Supplementary Table S13**.

One non-synonymous SNP, located on gene Jcr4S03126.20 and identified by KEGG analysis, was belonging to two pathways (sesquiterpenoid and triterpenoid biosynthesis, steroid biosynthesis) (**Supplementary Table S14**). Steroid hormones, which are important for plant growth and fruit development, are produced during steroid biosynthesis pathway via MAV (Mevalonate) pathway. On the other hand, triterpenoids and sesquiterpenoids, which have a significant role in biotic and abiotic stress response, are also generated via MVA pathway. These data could be supported by previous studies that identified region harboring variable changes involved in stress response (Zmienko et al., 2014; Ariani et al., 2016).

Efforts to identify EMS-induced changes by ddGBS, however, yielded no results.

## Discussion

Natural as well as induced mutations, which are responsible for genetic variation, are the raw material for plant breeding (Jiao et al., 2016). Recent studies showed the genetic diversity within *J. curcas* accessions to be narrow, resulting from genetic history, population bottlenecks, lack of evolutionary change or a high level of human-driven selection, which may have decreased the genetic diversity in *J. curcas* and increased its homogeneity (Doebley et al., 2006; Achten et al., 2010; Maghuly et al., 2015). Also, *J. curcas* is vegetatively propagated, which further reduces the genetic variation (Ambrosi et al., 2010). Further, the mating system plays a key role in homogeneity and low genetic diversity in *J. curcas*, being a mixed mating system through self- and cross-pollination, with high rates of full-sib seeds (Bressan et al., 2013; Negussie et al., 2014). On the other hand, because *Jatropha* species are often restricted to Isolated sites, where they adapted to a particular climate or soil types, the frequency of interbreeding is low, which may have additionally reduced the genetic diversity (Alves et al., 2013). In such cases inducing genetic diversity by generating a mutant population from a uniform genetic background like *Jatropha* is a robust strategy to create novel variations for crop improvement in plant breeding programs and to connect genotypic variation to phenotypic diversity (Jiao et al., 2016).

For Jatropha, so far, only a few studies have applied physical and chemical mutagenesis for its improvement, e.g., different doses of γ-rays (5-50 Kr) were reported to change various morphological traits in *Jatropha*. Unfortunately, these studies were only verified in M_1_ populations, without any reverse genetic analyses (Dhakshanamoorthy et al., 2011; Dhillon et al., 2014). Also, different concentrations of EMS (1–4%) were used in *J. curcas* (Dhakshanamoorthy et al., 2011), but only random amplified polymorphic DNA (RAPD) markers were used to identify polymorphisms.

The effect of the artificial mutation can be detected by different approaches, like the observation of phenotypic variation (forward genetics) (Mohd-Yusoff et al., 2015), however, in some case, phenotypic variations are not visible, since they may affect physiological and biological processes. For examples, in EMS-induced *Arabidopsis* mutants response to high temperature was affected through decreasing in ABA, and salicylic acid synthesis (Zhu et al., 2012), sensitivity to ABA was reduced in chemically mutated *L. japonica* (Biswas et al., 2009), in soybean TILLING an increasing in the oleic acid content was confirmed by identification of novel mutations in the FAD1, 2, and 3 genes (Dierking and Bilyeu, 2009) and in *Sorghum* the lignin content was decreased by mutation of the gene encoding caffeic acid *O*-methyltransferase (Xin et al., 2008).

In this case, the application of a cost-effective and high-throughput and efficient screening mutation (reverse genetics) method allowing to predict gene functions and to analyze the phenotypic consequences is advantageous. In fact, to identify novel traits in a large mutant population, and especially in plants without a complete reference genome, like *Jatropha*, a high throughput, cost-effective genetic screening technology is needed. Traditionally, TILLING is a popular method to identify causative SNPs in mutated population; however, it requires significant time and effort to design specific primers for selected genes (Sidhu et al., 2015). Next-generation sequencing experiments based genotyping is routinely used to find rare and novel variation and to detect mutations in various plant species (Sidhu et al., 2015). Among different reverse genetic approaches, GBS showed to be an efficient method to identify a mutation without prior knowledge of the target genes. It also proved to be a suitable method to determine SNPs and InDels, functional genes and the allele within a reasonably short period of time (Ariani et al., 2016). Therefore, for the first time, in the current study, the application of sequencing-based genotyping approaches was tested for the evaluation of vegetatively propagated *J. curcas* mutated by EMS.

During the experimental and data analyses process, we found that although the amounts of DNA per sample were equal, the number of generated reads for each sample and for each site was different, which also previously reported by (Elshire et al., 2011; Mascher et al., 2013; Liu et al., 2014; Sidhu et al., 2015). This could result in missing data and therefore has a significant effect on data analyses (Jiang et al., 2016). To overcome this limitation, it was suggested (Harloff et al., 2012) to add additional filter, use paired-end reads, and/or using alternative method to increase the site coverage and to generate more consistent results by introducing two enzymes like ddGBS (Peterson et al., 2012; Poland et al., 2012). In this case, selection and evaluation of the effect of the REs is critical, especially for plants with a high degree of DNA methylation like *J. curcas* (Yi et al., 2010). Interestingly, obtained result in the current study showed that the selected enzymes for ddGBS were not consistent with *in silico* simulation, since ddGBS analyses obtained lower numbers of fragment and SNPs compared two nGBS (**Supplementary Table S15**), resulting from the high frequency of DNA methylation. The reasons underlying the low number of fragments or missing data in ddGBS could be the following: (a) restriction sites are missing in samples because of biological cases or mutation (natural or induced mutation); (b) the DNA was not digested due to DNA methylation; (c) PCR was not as efficient for the specific fragment, and (d) low coverage of particular position, because of sequencing setup. However, the small number of reads and SNPs in the current study could be explained by the occurrence of high amounts of CpNpG in the *Jatropha* genome. In a survey based on methylation-sensitive fluorescence, (Yi et al., 2010) reported that more than half of the CCGG site found in *J. curcas* were methylated. In fact, most of the genetic variation reported for this species was shown to be mostly epigenetic (Carels, 2013). In plant systems, the most abundant context of methylation occurs within a C-G dinucleotide (CpG), usually symmetrically on both DNA strands. Since plants contain high amounts of CpG, *Pst*I, which is CpNpG sensitive, will generally be applied with other frequently cutting enzymes (4 cutters, e.g., *Msp*I) for GBS analyses. Therefore, for the selection of a suitable enzyme combination for ddGBS, it is helpful to carry out whole-genome bisulfite sequencing or to perform a small-scale pilot experiment with several combinations of restriction enzyme (Shirasawa et al., 2016).

On the other hand, the common problem of reduced representation sequencing methods like GBS are high error rates in distinguishing heterozygous and homozygous individuals, which can conceal the identification of true variation (Nielsen et al., 2011; Sidhu et al., 2015; Jiang et al., 2016). To identify variation induced by EMS, we added an additional set of filter, by using the variants that all wild accessions, containing similar genetic background, had in common.

We also found that the mutation spectrum encountered in the current study was prevalently G/C to A/T transitions (60% of base changes), which is in agreement with research carried out on barley (70%) (Caldwell et al., 2004), rice (70%) (Till et al., 2007), *Lotus japanicus* (65%) (Mohd-Yusoff et al., 2015) and tomato (60%) (Minoia et al., 2010). Only in *Arabidopsis* (99%) and banana (100%) a higher frequency of G/C to A/T base changes were identified. Further, besides G/C to A/T transitions also transversions were found in EMS mutated *Jatropha*, which represent one-third of the total substitutions. It was previously suggested, that they should not be removed from a mutation study since they could be a causative mutation for a trait of interest (Mohd-Yusoff et al., 2015). As described before, causative mutations in coding regions can have either a synonymous or non-synonymous effect, the later disrupting protein-coding sequences (Sikora et al., 2011). The identification of non-synonymous effects, as selected for functional annotation analyses, associated with phenotypic variation is of crucial importance.

Regardless of the method used for SNP detection, it is necessary to dissociate genotypic heterogeneity or chimeras, which occurs at the time of mutagenesis, to identify desirable genetically homogenous mutants. Chimerism normally is eliminated through sexual propagation by self-pollination of the mutated population (M_1_) to produce the M_2_ or higher populations (Comai and Henikoff, 2006). In the current study, the reduction of chimeras was achieved through vegetative propagation of apical as well as lateral meristems. Since previous studies on the mutant population of banana suggested that most chimera should be dissolved when reaching M_1_V_3_ and M_1_V_4_ populations (Jankowicz-Cieslak et al., 2012), it was considered that M_1_V_6_ sibling should be free of chimerism.

## Conclusion

In conclusion, a total of 96 M_1_V_6_ plants (wild-type and induced mutation populations) were selected, and the frequency and the effect of EMS treatments were compared using both nGBS and ddGBS analyses. As reverse genetic approaches, they represent an efficient method for discovery of the induced mutation in a large population with a large number of genes associated with phenotypic variation. Obtained data showed that allelic variations and *in silico* analysis of gene functions could be rapidly identified in mutant populations using nGBS and ddGBS. The results also confirmed the existence of high genome methylation in *J. curcas*, which could be responsible for its genome plasticity and ability to survive in different climate conditions. EMS-induced mutants exhibited a wide range of phenotypic variation, especially in leaf and stem architecture, which depends on the concentration of the applied mutagen (Maghuly et al., 2017).

A significant result of this study was to understand that the handling of GBS data is more difficult and more challenging than the traditional TILLING strategy in mutated plants, which is routinely used for reverse genetic analyses. Also, since it is difficult to get full coverage of potential protein-coding regions among all samples by these methods, it was also difficult to perform a study of average mutation density. Further, since the *Jatropha* genome sequence is incomplete and therefore contains missing genes, alignment and variant analysis of target sequence reads is challenging to perform and interpret. Thus, providing a complete *Jatropha* reference genome sequence with high quality should be a priority for any breeding program. However, increasing accessibility of high-throughput technology with decreasing cost of sequencing. Although in our data the ddGBS method showed low-density SNP markers and low genotyping accuracy, using suitable methylation sensitive or insensitive restriction enzymes will give the opportunity to use GBS with higher site coverage and lower genotyping error. At last, continuously vegetatively propagated tissue is a fast, efficient and accurate method to dissolve chimeras, especially for long-lived plants like *J. curcas*. Further, if the gene of interest contains a causative mutation, this should be validated.

## Author Contributions

FM and ML designed the whole study, carried out the all experiments, participated in data analyses, and wrote the final manuscript. SP and JK participated in data analyses and manuscript editing. All authors read and approved the final manuscript.

## Conflict of Interest Statement

The authors declare that the research was conducted in the absence of any commercial or financial relationships that could be construed as a potential conflict of interest.

## Footnotes

1 http://www.kazusa.or.jp/jatropha/

2 http://bio-bwa.sourceforge.net/

3 ftp://ftp.kazusa.or.jp/pub/jatropha/

4 https://github.com/ekg/freebayes

5 http://snpeff.sourceforge.net/

6 http://snpeff.sourceforge.net

7 ftp://ftp.kazusa.or.jp/pub/jatropha/

## Supplementary Material

FIGURE S1 Functional annotation of nGBS variants in genic regions in three GO categories (BP, MF, CC).

FIGURE S2 Functional annotation of EMS-induced SNPs identified by nGBS in genic regions in three GO categories (BP, MF, CC).

FIGURE S3 Functional annotation of ddGBS variants in genic regions in three GO categories (BP, MF, CC).

TABLE S1 List of 96 samples containing 14 non-mutated wild-type accessions, 82 mutated accessions, and their treatments.

TABLE S2 Distribution of nGBS reads in each sample and their proportion mapped to the reference.

TABLE S3 Annotation of nGBS variants on annotated genes and transcripts using SnpEff.

TABLE S4 Distribution of SNPs and InDels identified by nGBS in each sample.

TABLE S5 Functional annotation of nGBS variants in genic regions in three GO categories (BP, MF, CC).

TABLE S6 The nGBS variable alleles in genic regions assigned to KEGG pathways.

TABLE S7 Annotation of EMS-induced changes identified by the nGBS-based variant on annotated genes and transcripts using SnpEff.

TABLE S8 Distribution of EMS-induced SNPs identified by nGBS in each sample.

TABLE S9 Functional annotation of EMS-induced SNPs identified by nGBS in genic regions in three GO categories.

TABLE S10 Distribution of ddGBS reads in each sample and their proportion mapped to the reference.

TABLE S11 Annotation of ddGBS variants on annotated genes and transcripts using SnpEff.

TABLE S12 Distribution of SNPs and InDels identified by ddGBS in each sample.

TABLE S13 Functional annotation of ddGBS variants in genic regions in three GO categories (BP, FM, CC).

TABLE S14 The ddGBS variable alleles in genic regions assigned to KEGG pathways.

TABLE S15 Distribution of average number of reads and heterozygous SNPs for nGBS, ddGBS and each treatments.
