# Supplementary figures and images for: The Pattern and Distribution of Induced Mutations in J. curcas Using Reduced Representation Sequencing
Source: Front Plant Sci. 2018 Apr 23;9:524. doi: 10.3389/fpls.2018.00524 (PMC5944264; doi:10.3389/fpls.2018.00524)

BP

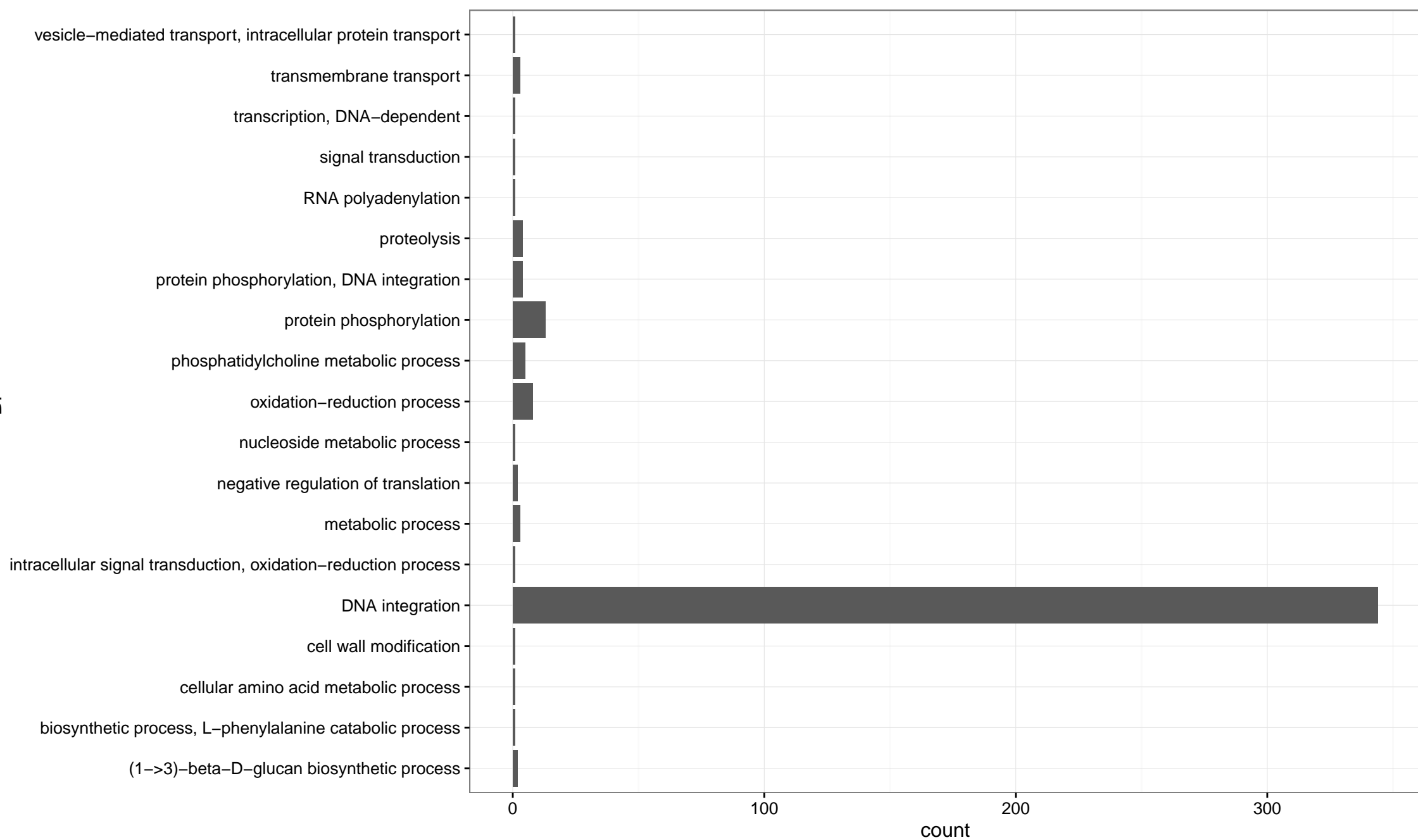

CC

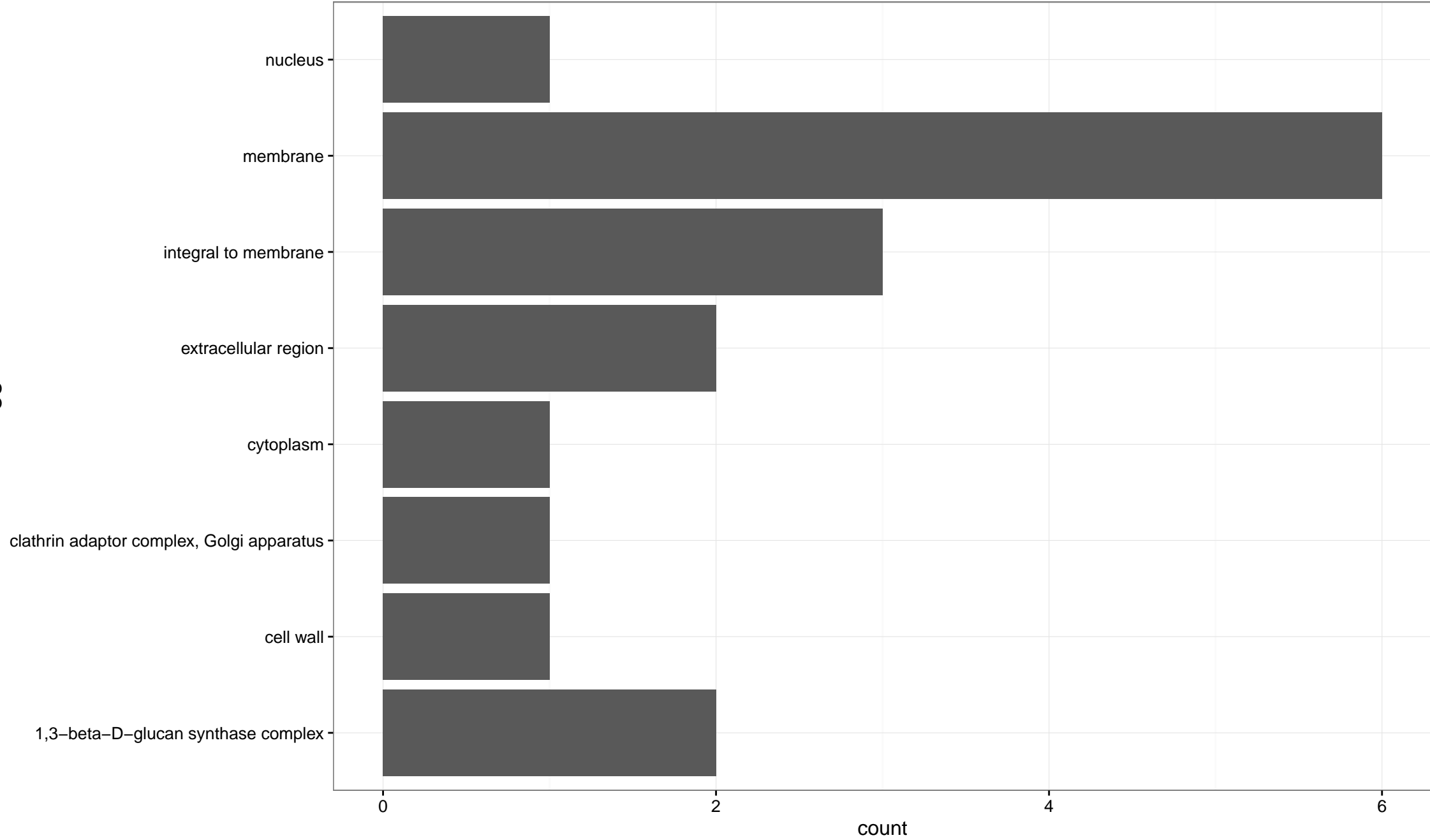

MF

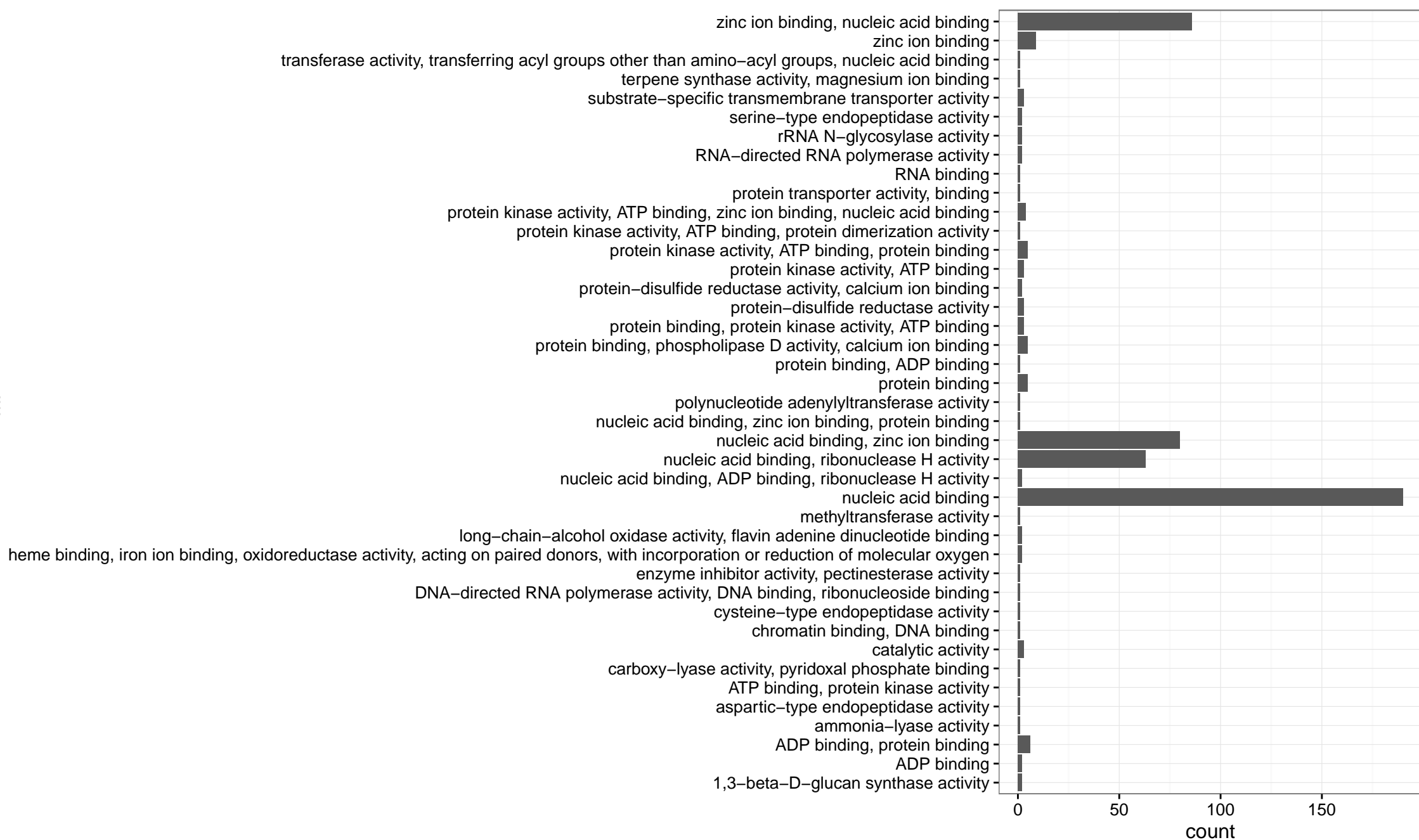

Supplement: FIGURE S1 — Functional annotation of nGBS variants in genic regions in three GO categories (BP, MF, CC). [file Image_1.PDF]

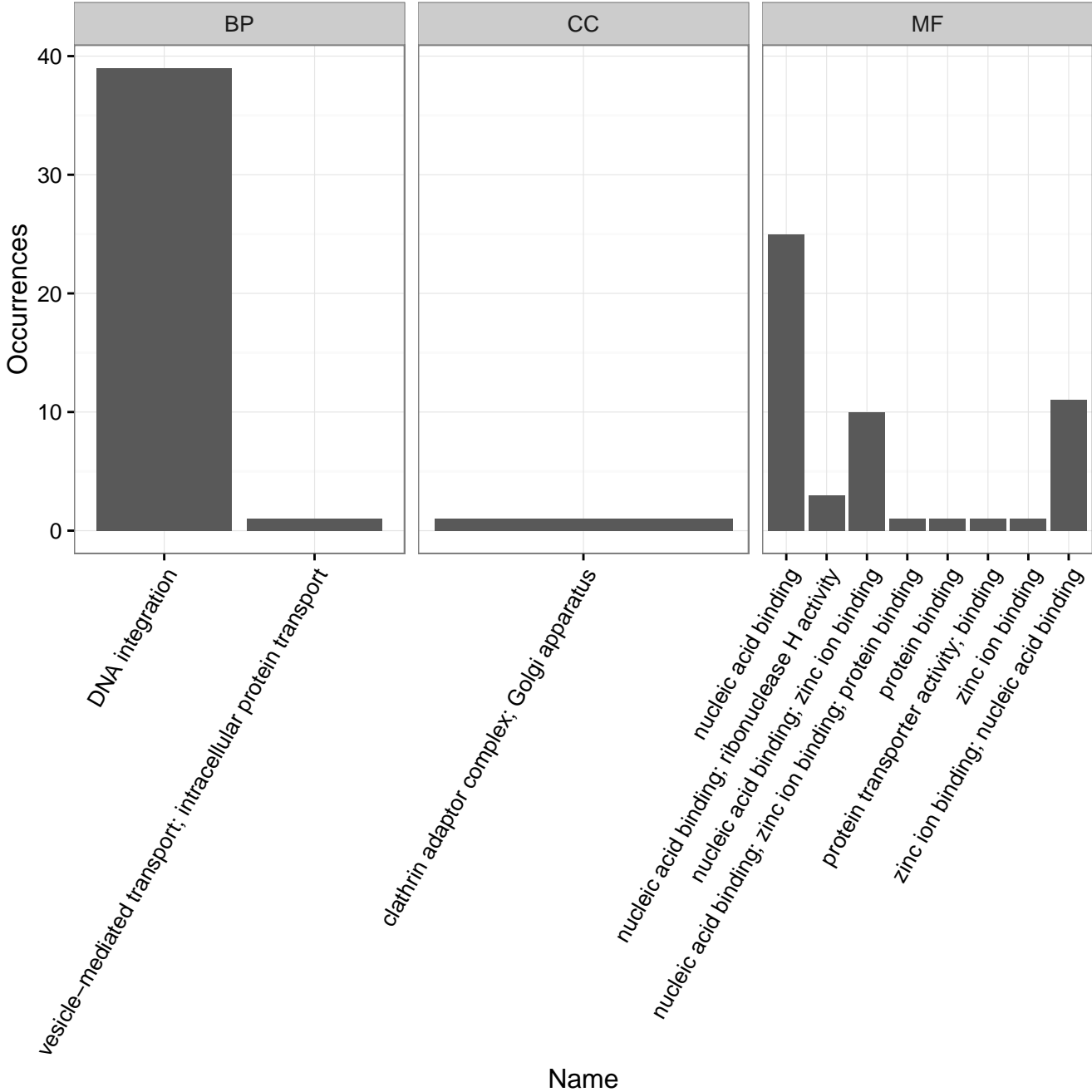

Supplement: FIGURE S2 — Functional annotation of EMS-induced SNPs identified by nGBS in genic regions in three GO categories (BP, MF, CC). [file Image_2.PDF]

BP

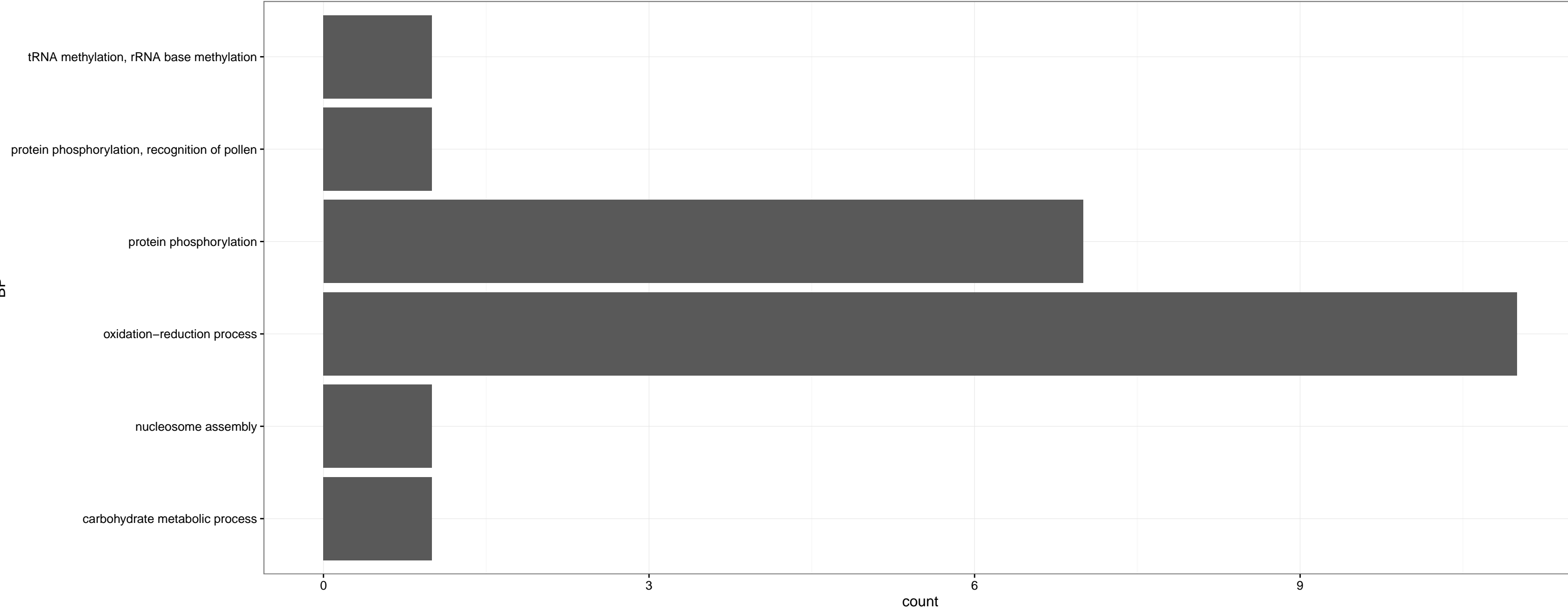

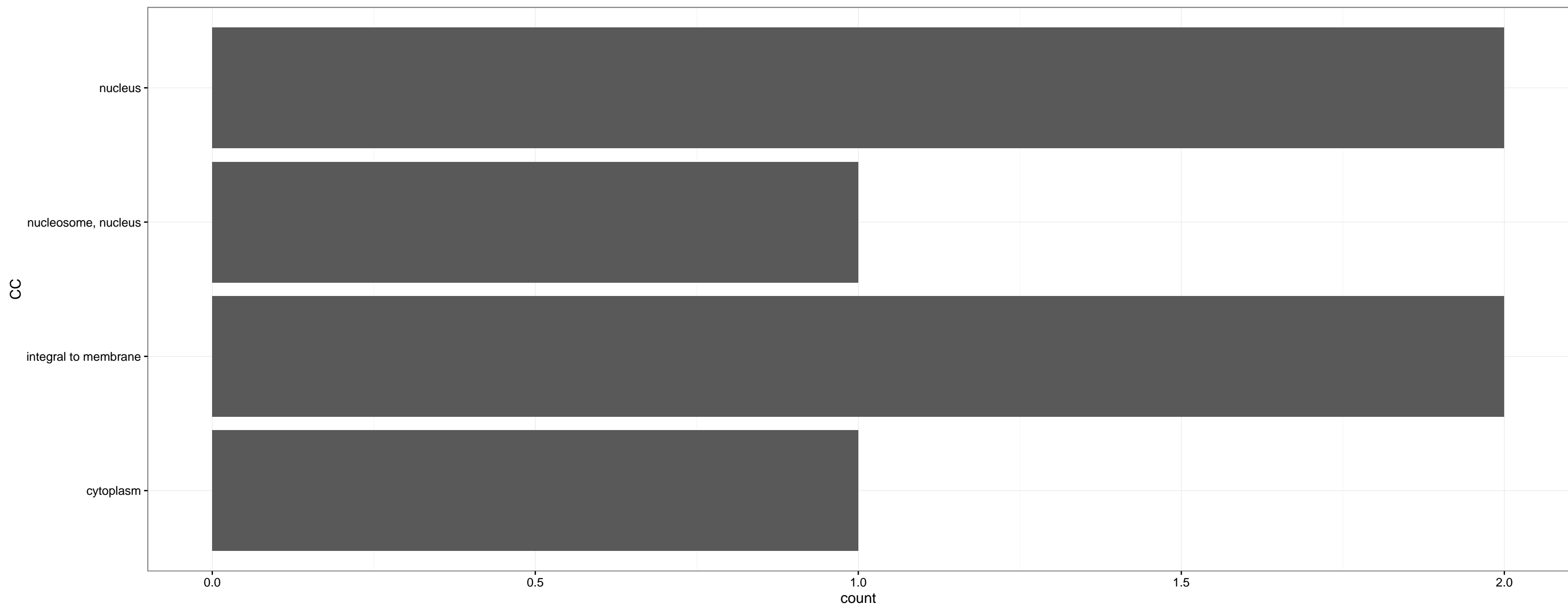

MF

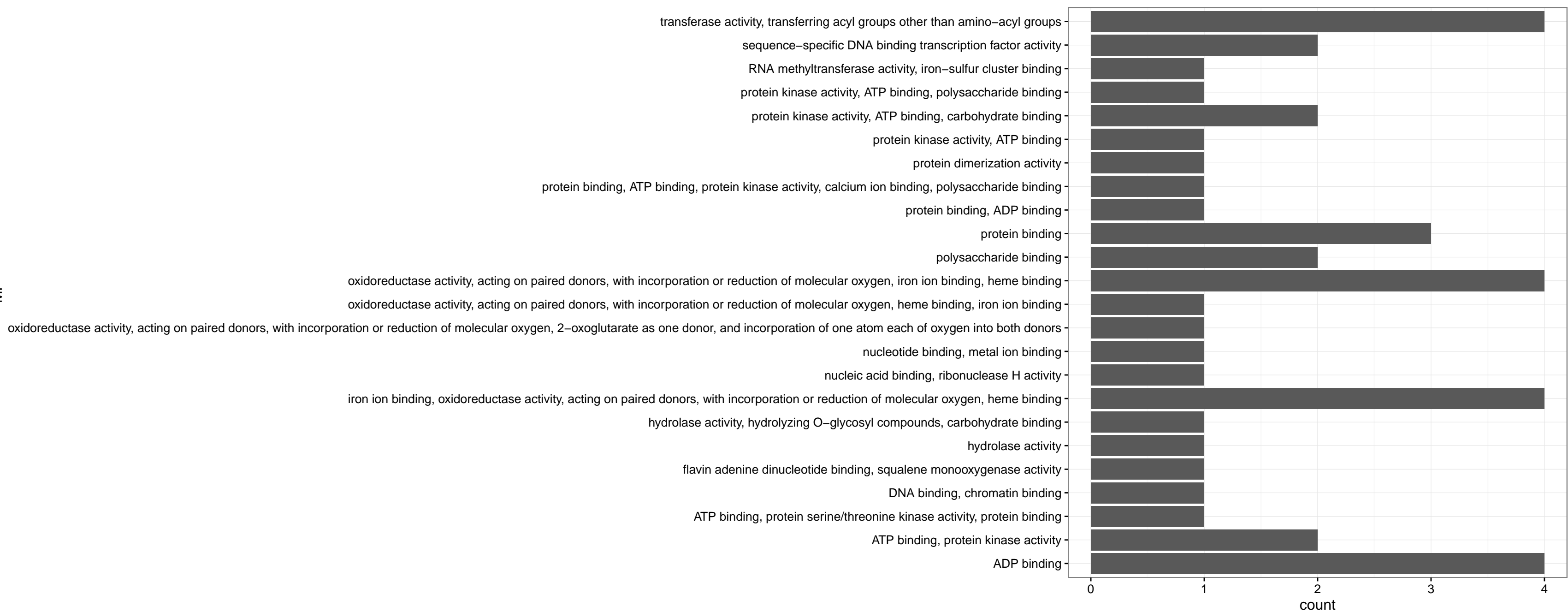

Supplement: FIGURE S3 — Functional annotation of ddGBS variants in genic regions in three GO categories (BP, MF, CC). [file Image_3.PDF]
